# Open questions: two challenges in chemical biology - chemical engineering and the science of diet

**DOI:** 10.1186/1741-7007-11-87

**Published:** 2013-07-30

**Authors:** Philip A Cole

**Affiliations:** 1 Department of Pharmacology and Molecular Sciences, Johns Hopkins University School of Medicine, Baltimore, MD 21205, USA

## 

A respected scientist was once asked what research project he thought he would be working on in five years. He responded that if he knew the answer, he would be working on it now. As the great New York Yankee baseball catcher Yogi Berra said, “It’s difficult to make predictions, especially about the future.” It is thus with considerable trepidation that I offer a short discussion about two research topics at the chemistry/biology interface that I believe may offer opportunities for major advances: protein chemical engineering and chemoprevention.

Understanding protein dynamics, relating structure and function, and deciphering the specific contributions of post-translational modifications has attracted intense effort but has yielded slow progress. Part of the challenge has been the limitation of the genetic code and the natural 20 amino acids. While making recombinant proteins has become relatively routine, the inability to introduce biophysical probes, isotopic labels, unnatural amino acids, and post-translational modifications site-specifically into proteins has been a major challenge historically. Recently developed methods for the chemical manipulation of proteins using semisynthetic strategies and unnatural amino acid mutagenesis promise to be increasingly powerful in the analysis of protein structure and function. Exploitation of inteins, sortase, and chemoselective modification reactions have already been valuable in modifying enzymes and histone proteins for the introduction of a wide range of post-translational modifications and fluorescent probes. Unnatural amino acid mutagenesis by the development of evolved aminoacyl tRNA synthases and nonsense suppression approaches have produced a range of chemically altered proteins. As synthetic biology develops organisms with artificial genomes lacking particular nonsense codons, it should be possible to create cells and animals with a wide range of chemical modifications. Such techniques are likely to be of special utility for membrane proteins, which are often very complicated to study. As these protein chemical methods are adapted to cellular and *in vivo* experiments, they should be transformative in illuminating the contributions such proteins make to cell signaling and gene regulation.

Another emerging area for chemistry/biology interface research is in disease prevention using natural products and their analogs. The search for, and application of, chemical substances in dietary plants has garnered increasing attention among clinical and basic science investigators in recent years. Whereas herbal products have had a rich history as a source of pharmaceuticals in Chinese and Western medicine, the focus on prevention rather than treatment is gaining currency with researchers and the general public. As health care costs rise inexorably, massive late stage expenditures in the last year of life to stave off advanced illness are unsustainable. It is generally recognized that identifying a health problem at an early phase offers the best chance for successful and cost-effective therapeutic intervention. In this light, chemoprevention through dietary adjustment is an attractive public health strategy. However, there has been a justified skepticism surrounding research in this area, which has sometimes lacked rigor and molecular detail. To move this field from the phenomenological to the mechanistic, defining key molecular pathways that are affected by chemoprevention compounds is critical. Such has been elegantly demonstrated with the broccoli compound sulforaphane and its interactions with the Keap1/Nrf2 anti-oxidant response pathway. Dozens of clinical trials are underway for myriad conditions to determine if broccoli extracts rich in sulforaphane can protect against cancer, inflammatory diseases, and photo-irradiation-induced skin damage. Going forward, there are likely to be many molecular mechanisms yet to be discovered that provide opportunities for chemoprevention. There is also much to still be learned in nutritional science and metabolomics that could have a major impact on public health.

